# Assessing the robustness of an artificial intelligence segmentation model for quantitative cardiovascular magnetic resonance imaging across cardiac phenotypes

**DOI:** 10.1007/s10554-025-03596-3

**Published:** 2025-12-26

**Authors:** Hadil Saad, Clemens Ammann, Thomas Hadler, Yashraj Bhoyroo, Philine Reisdorf, Jana Veit, Teodora Chitiboi, Jens Wetzl, Christian Geppert, Jeanette Schulz-Menger

**Affiliations:** 1 Charité – Universitätsmedizin Berlin, corporate member of Freie Universität Berlin and Humboldt Universität zu Berlin, Berlin, Germany; 2 Working Group on CMR, Experimental and Clinical Research Center, Max Delbrück Center for Molecular Medicine in the Helmholtz Association and Charité – Universitätsmedizin Berlin, Berlin, Germany; 3 Department of Cardiology and Nephrology, Helios Hospital Berlin-Buch, Berlin, Germany; 4 DZHK (German Centre for Cardiovascular Research), partner site Berlin, Berlin, Germany; 5 Siemens Healthcare GmbH, Hamburg, Germany; 6 Research & Clinical Translation, Magnetic Resonance, Siemens Healthineers AG, Erlangen, Germany

**Keywords:** Cardiovascular magnetic resonance, Artificial intelligence, Cardiac phenotypes, Segmentation, Tissue characterization, Cardiac function

## Abstract

**Purpose:**

To introduce an artificial intelligence-based cardiovascular magnetic resonance segmentation algorithm (Nick) for automated quantification of function and parametric mapping across cardiac phenotypes reflecting clinical routine.

**Methods:**

Nick was compared to manual gold standard (GS) segmentations in 359 multi-centre cases at 1.5T and 3T, consisting of 104 healthy individuals and 255 diseased patients with various cardiac phenotypes. Left and right ventricular (LV, RV) volumes and LV mass (LVM) were derived from short-axis segmentations. For parametric mapping, the LV myocardium was segmented to quantify T1 and T2 relaxation times. Statistical analysis comprised mean differences, correlation coefficients (R²), Bland-Altman analysis, tolerance range assessments, and paired boxplots. The number of slices and contours requiring manual correction was estimated based on slice-level differences.

**Results:**

Nick demonstrated high agreement with the GS for LV and RV volume estimations (R²≥0.93) and LVM quantification (R²=0.86). For the ejection fractions, correlations were slightly lower (R²=0.85/0.72 for LV/RV) with small mean differences (+ 1.14%/-2.48% for LV/RV). T1 and T2 mapping values showed excellent agreement with manual reference values (R²≥0.92) and minimal biases (-1.64/0.14 ms for T1/T2). Nick underestimated LV volumes at end-diastole (-4.48 ml) and end-systole (-3.28 ml) as well as the RV end-diastolic volume (-5.14 ml) and stroke volume (-6.75 ml). Nonetheless, tolerance testing for mean deviations revealed clinically acceptable biases for all comparisons, and less than two slices per case required correction on average.

**Conclusion:**

Comparison to expert segmentations revealed robust performance of Nick in routine clinical cases with variable pathology, supporting its future integration into clinical workflows.

**Graphical Abstract:**

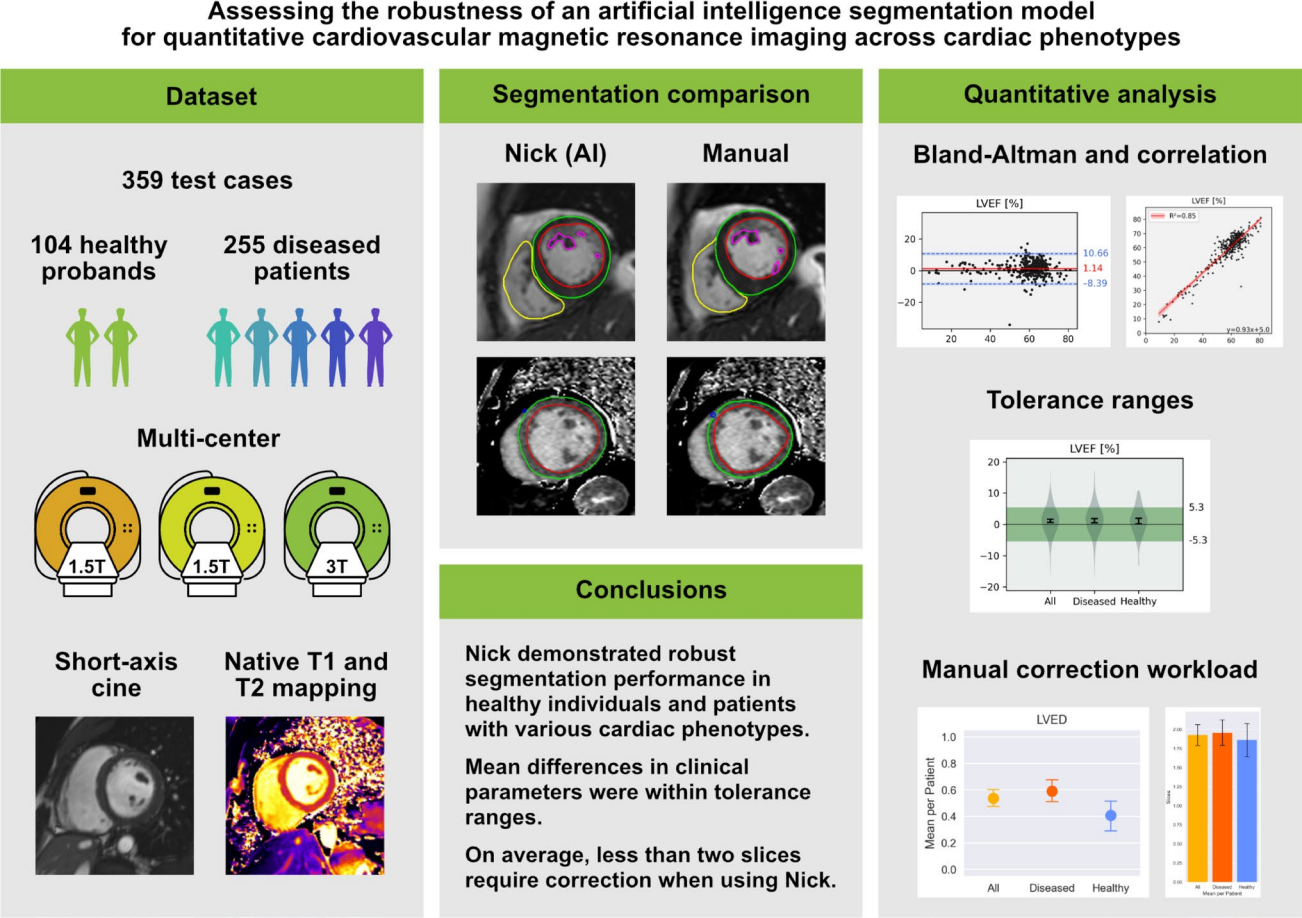

**Supplementary Information:**

The online version contains supplementary material available at 10.1007/s10554-025-03596-3.

## Introduction

Cardiovascular magnetic resonance (CMR) provides accurate, reproducible measurements of cardiac structure, function and myocardial tissue differentiation [1]. Cine imaging enables the assessment of quantitative parameters including ejection fraction (EF), stroke volume (SV), end-diastolic and end-systolic ventricular volumes (EDV, ESV) and left ventricular mass (LVM) [2], while parametric mapping uniquely allows for non-invasive tissue characterization by T1 and T2 relaxation times [3, 4].

As CMR adoption grows in clinical and research settings, the resulting increase in data volume and complexity [5–7] poses challenges for manual analysis, which is time consuming and subject to observer variability [8]. In recent years, research into automated artificial intelligence (AI)-based segmentation tools has grown rapidly, offering a promising solution for faster and more consistent quantification of cardiac structure and function [9]. Deep learning models, particularly convolutional neural networks, have demonstrated substantial efficiency gains [10]. However, their performance often drops in real-world clinical settings due to variability in image quality, acquisition protocols, artifacts, and the presence of different phenotypes with diverse anatomical features [11–13]. For instance, left ventricular hypertrophy (LVH) or dilated cardiomyopathy (DCM) present with distinct structural and functional changes, including increased wall thickness or chamber dilation and impaired systolic function [14, 15].

A lack of data heterogeneity in training and test cases can reduce algorithm performance and limit generalizability in real-world clinical scenarios [11, 16]. Beyond accuracy, trust in AI models depends on consistent performance across diverse clinical settings. In particular, many existing deep learning approaches for CMR segmentation rely predominantly on slice-wise short-axis (SAX) processing and simplified heuristic rules for defining ventricular basal slices. These strategies frequently fail in the basal and apical regions of the ventricles: a detailed analysis of AI methods in the ACDC segmentation challenge reported anatomically implausible segmentations in about 1.6 slices per subject [17]. Such failure modes can impact derived clinical parameters and undermine user confidence in automated analysis.

This study introduces the research algorithm Nick, an AI model for SAX ventricular segmentation of cine imaging and parametric mapping, and comprehensively assesses its generalizability and robustness across healthy individuals and patients with varying cardiac phenotypes.

## Methods and materials

### Dataset

The retrospective test dataset consists of 359 adult cases from multiple sites using scanners of the same vendor at 1.5T and 3T. It comprises 104 healthy volunteers with normal cardiac structure and function, and 255 patients representing the diseased subcohort. None of the cases were used for training or validating the algorithm. Physician-assigned clinical diagnoses in the diseased group encompassed a broad spectrum of cardiovascular disease, including ischemic and non-ischemic cardiomyopathies, cardiac arrhythmias, hypertension, inflammatory heart disease, valvular disease, and cases with cardiac involvement of systemic disease. Diagnoses are given in Supplementary Table 1.

For subgroup analysis, the diseased cohort was further characterized by two distinct cardiac phenotypes: LVH, comprising amyloidosis and hypertrophic cardiomyopathy, and DCM. These were selected because they represent particularly characteristic structural alterations that may be challenging for automated segmentation by AI methods.

Variations in scanning protocols and exclusions due to artifacts or incomplete cardiac coverage led to diverging numbers of available imaging sequences per case (Table 1).

**Table 1:** Cohort overview

	Overall	Healthy	Diseased
Number of cases	359	104	255 (32 LVH, 36 DCM)
Including short-axis cine	350	103	247 (32 LVH, 33 DCM)
Including native T1 mapping	279	96	183 (23 LVH, 22 DCM)
including T2 mapping	249	94	155 (22 LVH, 23 DCM)

LVH: left ventricular hypertrophy; DCM: dilated cardiomyopathy

### CMR protocol

CMR images were acquired across multiple centres using Siemens 1.5T (MAGNETOM Avanto^Fit^, Avanto, Aera, Sola) and 3T (MAGNETOM Skyra^Fit^) scanners with varying protocols (Siemens Healthineers, Forchheim, Germany) and different levels of experience in CMR. SAX cine imaging used a gated balanced steady-state free precession sequence from base to apex (slice thickness 7–10 mm, interslice gap 0.0–1.6 mm). Overall, 279 cases included native T1 mapping and 249 cases included T2 mapping in one midventricular and one basal SAX slice. T1 mapping was based on a motion-corrected modified look-locker inversion recovery 5(3)3 sequence, while T2 mapping was based on a true fast imaging with steady-state precession sequence in scans at 1.5T and a fast low-angle shot sequence in 3T scans [3].

### Manual image segmentation

A comprehensive visual quality check was carried out for all cases by an experienced reader (more than 2,000 cases of experience, Society for Cardiovascular Magnetic Resonance [SCMR] Level II). After exclusion of images with artifacts, the images were manually segmented using a dedicated commercially available software (cvi42, versions 5.13.7 and 5.11.1, Circle Cardiovascular Imaging, Calgary, Canada). Gold standard (GS) annotations were conducted following the recommendations of the SCMR [1] and were reviewed by experienced readers with SCMR level III. Cases with disagreements were resolved by a consensus read. For cine imaging, segmentation was performed at end-diastole (ED) and end-systole (ES) with individual phase selection for each ventricle. Left ventricular (LV) papillary muscles were excluded from the blood pool and included in the calculation of LVM using a dedicated contour. For the right ventricle (RV), the basal slice was determined by checking for the presence of the pulmonary valve or the enlargement of the RV cavity during diastole to avoid contouring the right atrium. RV trabeculae and papillary muscles were included in the blood pool volume. For T1 and T2 mapping, endo- and epicardial contours of the LV myocardium were delineated in two slices (basal and midventricular), avoiding the inclusion of pericardium, blood pool or RV.

### Automatic image segmentation (Nick)

Nick automatically segmented the LV and RV endocardium, LV papillary muscles as well as the LV myocardium in all cardiac phases for SAX cine images. We designed a deep neural network based on the U-Net architecture [18] with five densely connected blocks [19] in both the encoder and decoder. The network takes individual 2D SAX cine frames as input and produces a multichannel output. Post-processing keeps only the largest connected component in each 2D slice for LV blood pool, RV blood pool, and LV myocardium. In addition, a 3D convolutional neural network was trained to predict the SAX slices containing the basal and apical regions of the ventricles. This network takes the full stack of SAX ED frames as input and predicts the base and apex positions separately for LV and RV. A separate segmentation network was trained on 2- and 4-chamber long-axis (LAX) cine images to segment atria and ventricles. These LAX segmentations are not used directly for volumetric computation, but to guide SAX segmentation. Specifically, they are used to compute the ED-to-ES displacement of the mitral and tricuspid valve planes. To account for systolic shortening, the LV and RV basal slice positions predicted at ED by the 3D network are adjusted at ES using the valve-plane displacement derived from the LAX segmentations. Any SAX segmentations lying outside the predicted base-to-apex slice interval are automatically removed.

For segmentation of T1 and T2 mapping images, we used a pretrained Dense U-Net [20] which was further finetuned to predict the right ventricular insertion point and segmentation of the LV myocardium. Network training is detailed in the Supplementary Information.

For comparison with the GS, segmentations at ED and ES were selected based on maximal and minimal ventricular volumes. The selected phases were allowed to differ between LV and RV, as well as from the expert selection. All processing was automatically performed directly on DICOM images, and no manual intervention was required for any of the cases in this study.

### Qualitative analysis

To evaluate the quality of Nick’s segmentations, all cases were visually inspected by two experienced readers. Segmentations were assessed for the accuracy of endo- and epicardial LV delineation, the papillary muscles, and endocardial RV delineation to ensure proper anatomical boundary detection. Specific attention was given to the phase selection for ED and ES. Qualitative evaluation criteria included identifying any major discrepancies, such as nonsensical contours completely disregarding cardiac anatomy, as well as over-segmentation or overlooked segmentations in basal and apical slices.

### Quantitative analysis

The comparison between Nick and GS segmentations was performed using the open-source medical image analysis platform *Lazy Luna* [21]. The software allows the similarity assessment of contours and provides clinical parameter comparisons. The following parameters were calculated from DICOM images and contours in *Lazy Luna*: left and right ventricular EDV (LVEDV, RVEDV), ESV (LVESV, RVESV), SV (LVSV, RVSV), EF (LVEF, RVEF), LVM at ED, and global native T1 and T2 relaxation times. Figure creation and additional statistical analyses were carried out in Python 3.12. For clinical parameter analysis, the agreement between Nick and GS was evaluated using correlation coefficients (R^2^) and Bland-Altman analysis to assess systematic bias. To determine the clinical acceptability of mean differences between GS and Nick, acceptance testing was performed. The bias for each parameter was considered acceptable if the 95% confidence interval lay completely within the limits of the respective predefined tolerance range established by intra-observer analysis in previously published studies [22, 23].

### Group analysis

Nick’s performance was assessed separately in healthy and diseased groups by comparing clinical parameters for LV and RV volumes, LVM, and T1/T2 relaxation times. Statistical significance was assessed using two-sided paired t-tests (significance level α = 0.05). Paired boxplots were used to visualize the distribution of quantitative parameters in both groups. Additionally, patients with LVH and DCM were analysed separately, focusing on differences in SV and EF to evaluate Nick’s performance across distinct cardiac phenotypes.

### Quantification of manual correction workload

To measure the number of potentially required manual corrections when using Nick, contour-specific limits were defined for segmentations. These represent tolerable deviations at the level of individual contours based on human interobserver variability. Cutoff values were defined from manual segmentations of 44 independent cases from two expert readers. A cutoff value of 1.96 standard deviations of the interobserver difference was defined for the absolute area difference, and a cutoff value of the median minus the median absolute deviation was defined for the Dice Similarity Coefficient (DSC). If the difference between the GS segmentation and Nick’s segmentation exceeded this limit for the absolute area difference and at the same time fell below the DSC threshold, the corresponding segmentation was considered to require manual correction. This combination captures relevant segmentation differences (absolute area difference) while omitting minor differences in large segmentations with significant overlap (DSC).

## Results

### Dataset

The study population, including demographics and characteristics (based on the GS quantifications) for the healthy and diseased groups, is summarized in Table 2. The diseased group was characterized by a lower proportion of women (39% vs. 59%), higher age (+ 17.6 years) and weight (+ 11.3 kg), reduced LVEF (−3.0%), and increased LVM (+ 31.9 g).

**Table 2:** Characteristic data of the cases in the cohort

		Total cases (*n* = 359)	Healthy group (*n* = 104)	Diseased group (*n* = 255)	*p*-value healthy vs. diseased
Basic demo-graphics	Age [years]	51.7 ± 17.7	39.2 ± 15.7	56.8 ± 15.8	< 0.001
	Height [cm]	173.9 ± 9.6	173.9 ± 9.0	173.9 ± 9.8	0.950
	Weight [kg]	79.9 ± 16.5	71.9 ± 12.6	83.2 ± 16.9	< 0.001
	Women	161 (45%)	61 (59%)	100 (39%)	
Basic characteristics	LVEF [%]	58.1 ± 12.3	61.1 ± 4.3	56.8 ± 14.2	0.003
	RVEF [%]	51.6 ± 8.7	52.5 ± 4.8	51.2 ± 9.9	0.197
	LVM [g]	100.8 ± 39.6	78.3 ± 24.8	110.2 ± 40.8	< 0.001
	Global native T1 [ms]	1035.6 ± 90.5	1001.3 ± 41.4	1053.5 ± 103.3	< 0.001
	Global T2 [ms]	48.2 ± 3.7	48.6 ± 2.5	47.9 ± 4.2	0.150

LVEF: left ventricular ejection fraction; RVEF: right ventricular ejection fraction; LVM: left ventricular mass

### Qualitative analysis

**Fig. 1:**
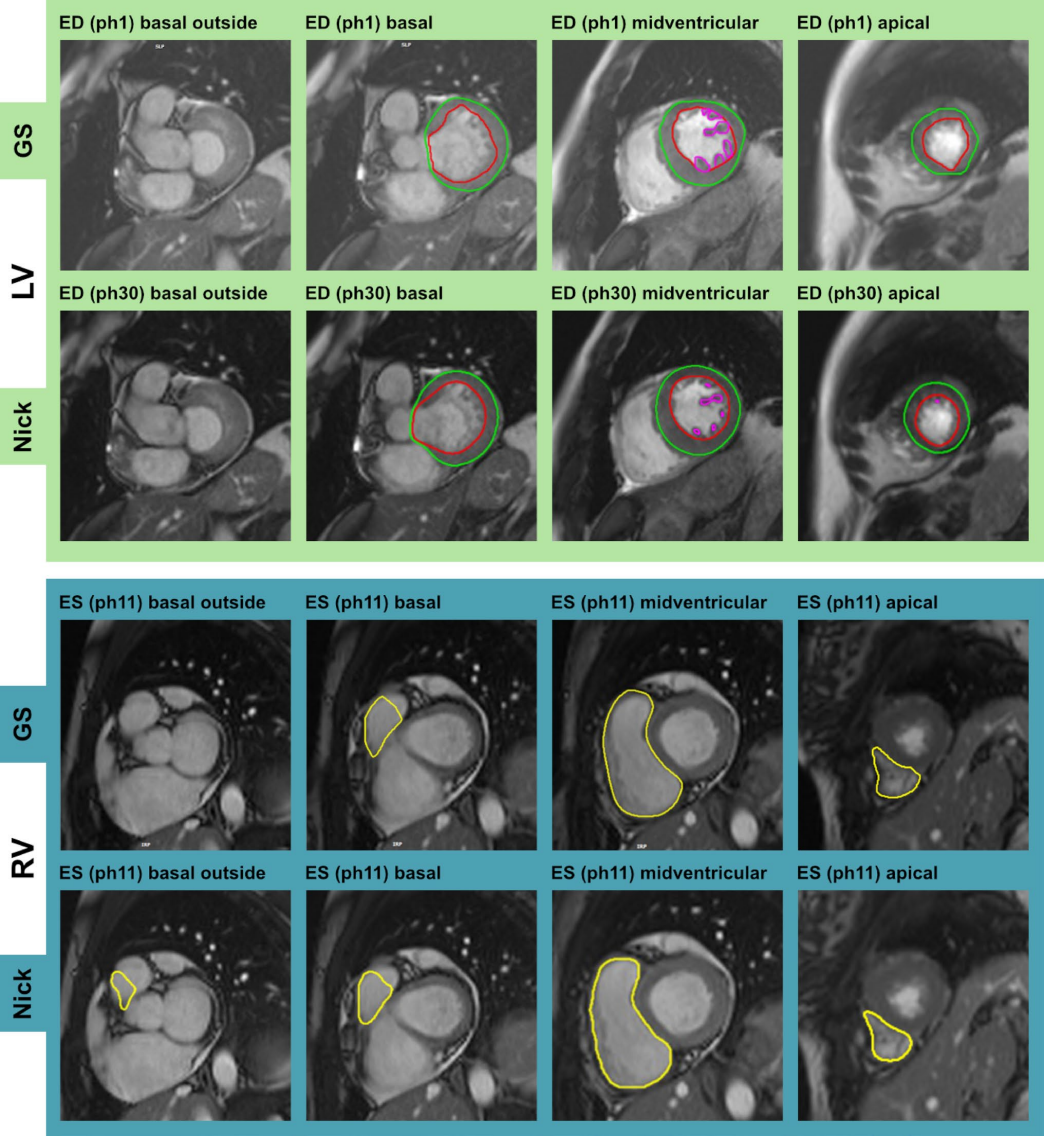
Two examples of Nick and gold standard (GS) segmentations in the short axis for the left ventricle (LV) and the right ventricle (RV). Selected cardiac phases (ph) for end-diastole (ED) and end-systole (ES) may deviate for Nick and GS

Nick processed all cases without technical failure, defined as the absence of segmentation output, incomplete processing, or the generation of severely distorted contours. Visual inspection of Nick’s contours showed no major discrepancies with the GS or nonsense contours completely disregarding cardiac anatomy. The automated selection of ED and ES cardiac phases by Nick closely matched expert selection. Figure 1 shows two examples of biventricular segmentations including epicardial, endocardial, and papillary muscle contours segmented by Nick and compared to GS annotations.

**Fig. 2:**
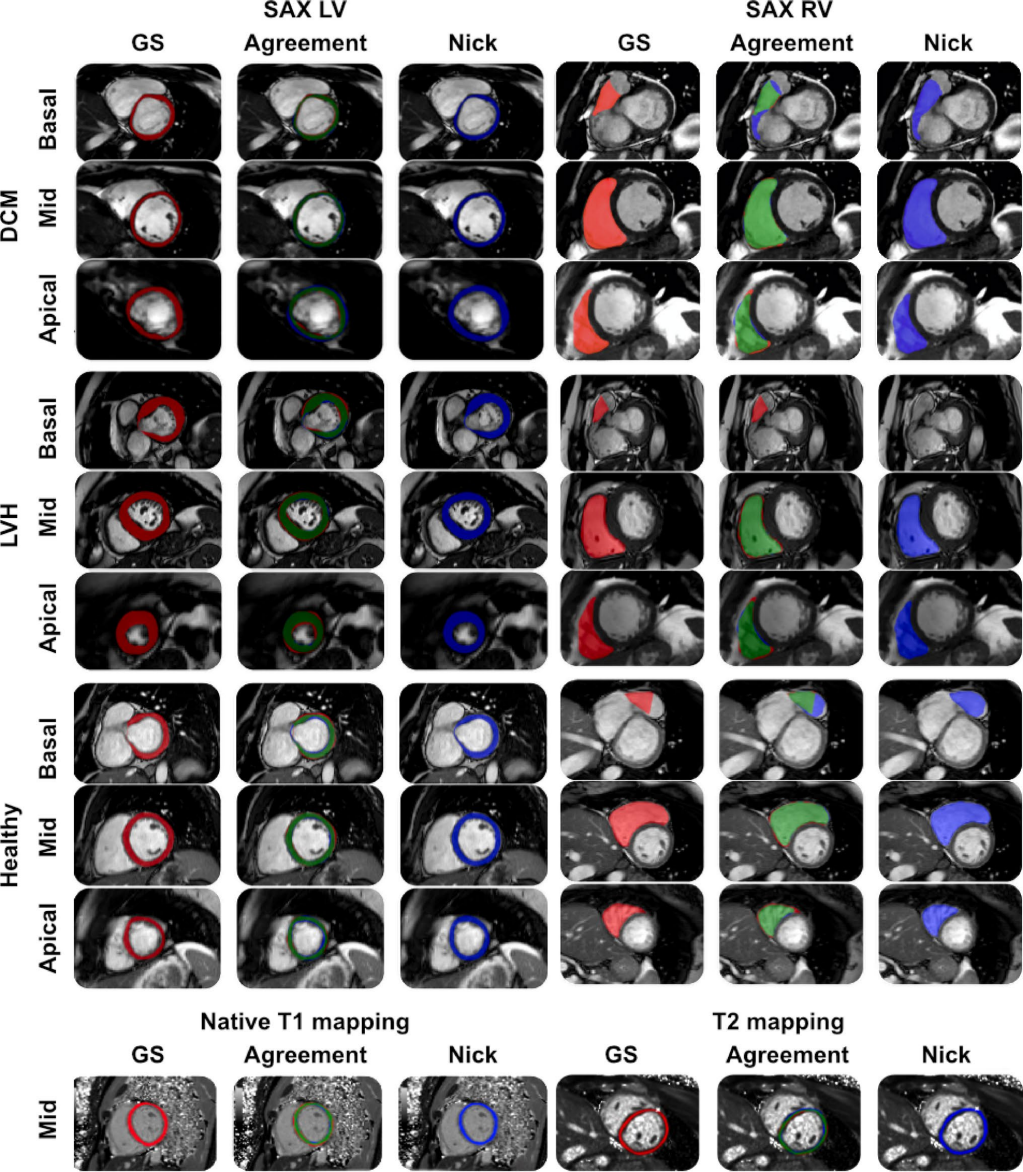
Comparison of Nick vs. gold standard (GS) segmentations across various cases using *Lazy Luna*. The panels “DCM” (dilated cardiomyopathy), “LVH” (left-ventricular hypertrophy), and “Healthy” each visualize short-axis (SAX) cine slices at basal, midventricular (mid), and apical positions. Red-shaded areas indicate GS segmentations, blue-shaded areas represent Nick’s segmentations, and green-shaded areas show their agreement. LV: left ventricle; RV: right ventricle

Figure 2 illustrates representative examples of Nick’s contours for LV and RV in comparison with GS contours using *Lazy Luna*. These examples illustrate Nick’s performance across different phenotypes, including DCM, LVH, and healthy cases, reflecting consistent segmentation despite morphological and pathological variation. T1 and T2 maps further demonstrate strong agreement between Nick and the GS. In basal and apical SAX slices, segmentation errors were noted, including over-segmentation or overlooked segmentations. However, these issues were infrequent, and most contours remained qualitatively acceptable.

### Quantitative analysis

#### Clinical parameter analysis

**Fig. 3:**
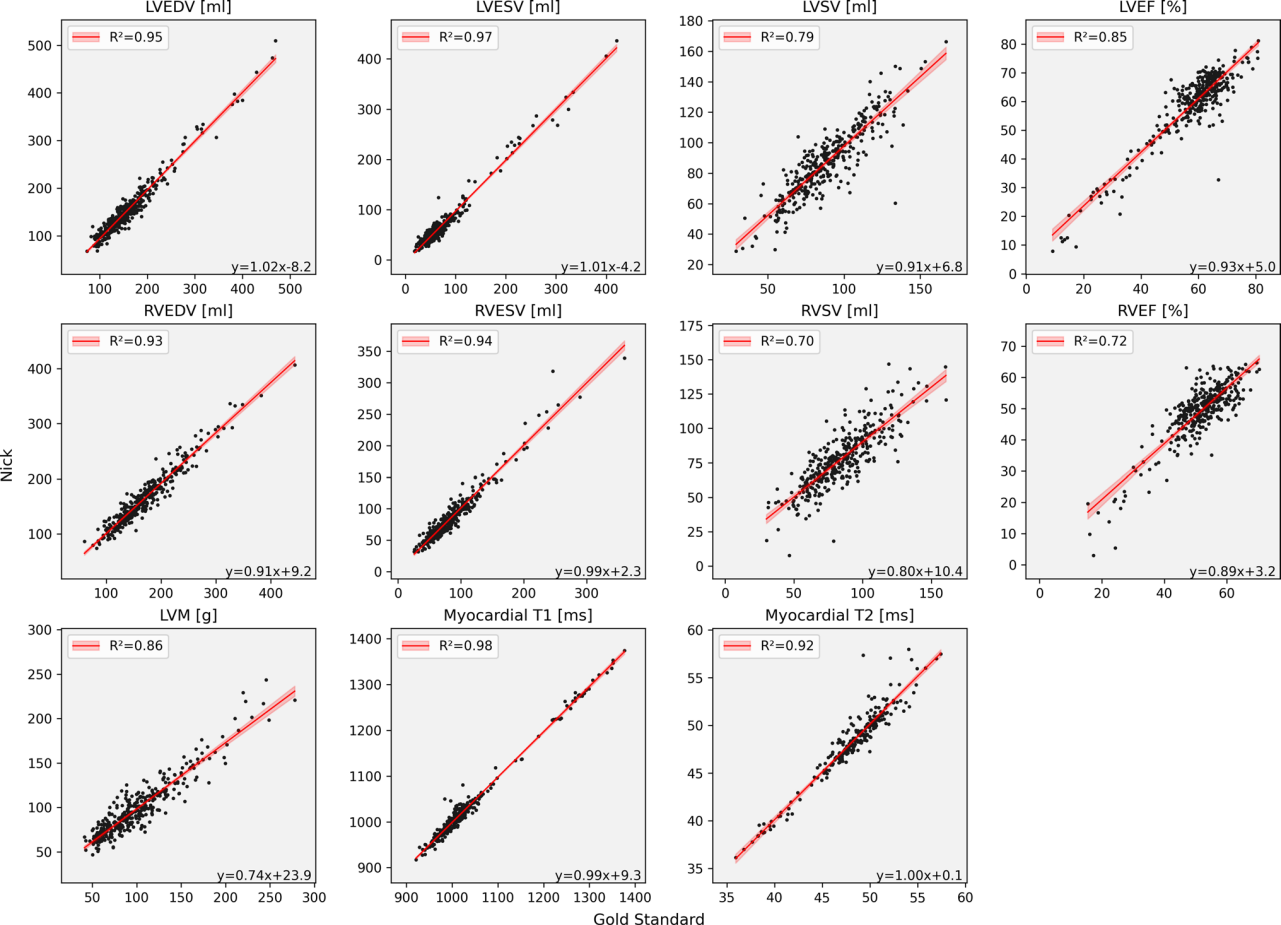
Correlation plots for right and left ventricular function parameters and parametric mapping in the overall cohort. LVEDV: left ventricular end-diastolic volume; LVESV: left ventricular end-systolic volume; LVSV: left ventricular stroke volume; LVEF: left ventricular ejection fraction; RVEDV: right ventricular end-diastolic volume; RVESV: right ventricular end-systolic volume; RVSV: right ventricular stroke volume; RVEF: right ventricular ejection fraction; LVM: left ventricular mass

Quantitative parameters derived from Nick’s LV segmentations showed high correlations with the reference values derived from GS segmentations (Fig. 3): LVEDV (R² = 0.95), LVESV (R² = 0.97), LVEF (R² = 0.85), LVSV (R² = 0.79), and LVM (R² = 0.86). For the RV, correlations with the GS were slightly lower: RVEDV (R² = 0.93), RVESV (R² = 0.94), RVSV (R² = 0.70), and RVEF (R² = 0.72). Parametric mapping values demonstrated strong correlations with the GS: Global T1 (R² = 0.98) and Global T2 (R² = 0.92). The selection of ED and ES showed excellent agreement with mean differences close to zero for LV (ED: 0.09 ± 0.91 cardiac phases; ES: 0.00 ± 0.87 cardiac phases) and RV (ED: −0.16 ± 0.92 cardiac phases; ES: 0.34 ± 1.11 cardiac phases).

**Fig. 4:**
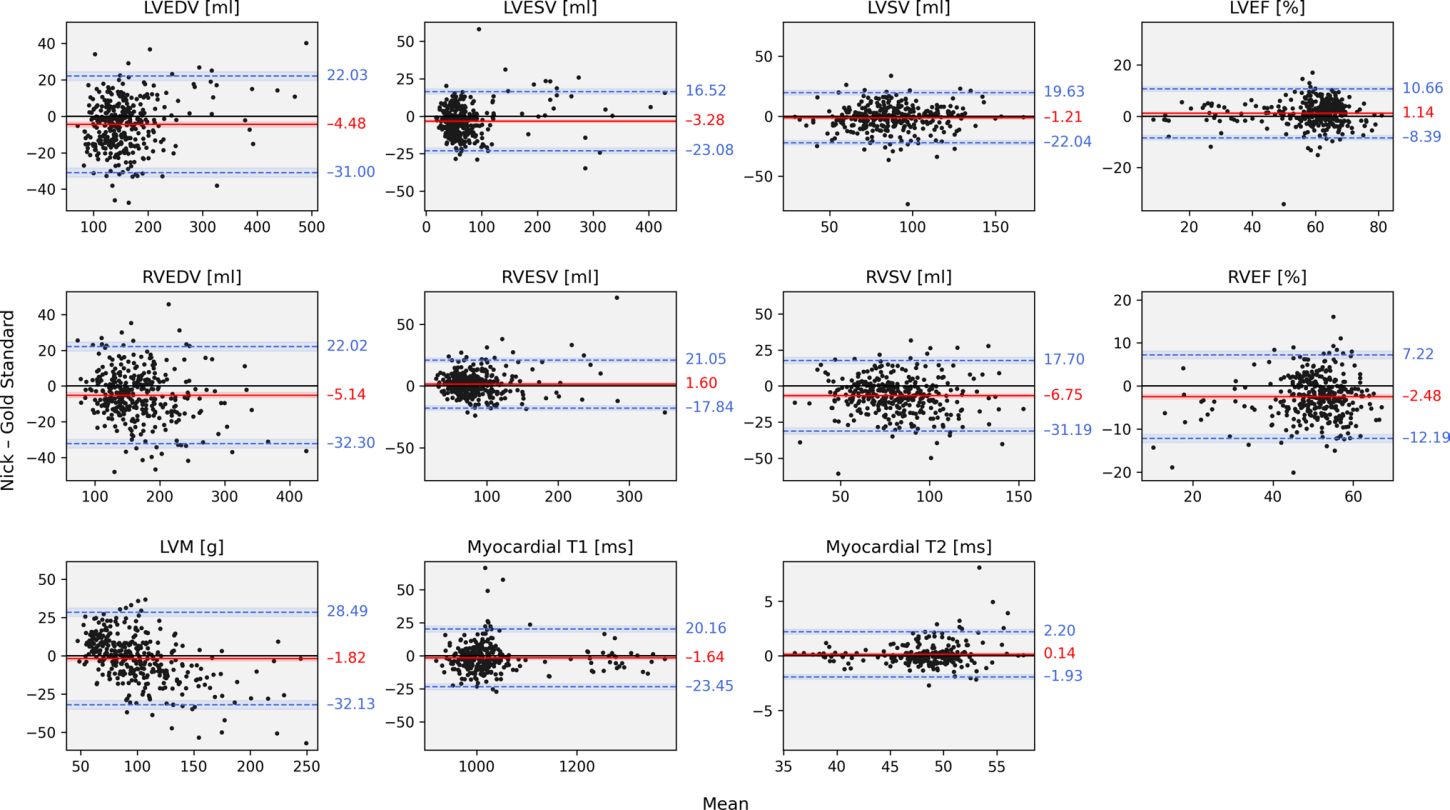
Bland-Altman plots for Nick and the gold standard (GS) in the overall cohort. The x-axis shows the average of Nick and GS, and the y-axis their difference (Nick - GS). The solid red line is the mean difference (bias) and the two dashed blue lines are ± 1.96 standard deviations from the mean difference. LVEDV: left ventricular end-diastolic volume; LVESV: left ventricular end-systolic volume; LVSV: left ventricular stroke volume; LVEF: left ventricular ejection fraction; RVEDV: right ventricular end-diastolic volume; RVESV: right ventricular end-systolic volume; RVSV: right ventricular stroke volume; RVEF: right ventricular ejection fraction; LVM: left ventricular mass

Bland-Altman plots are presented in Fig. 4. The analysis showed only moderate mean deviations (reported as mean ± standard deviation) between Nick and the GS evaluation with most data points falling within the limits of agreement. Due to consistently negative deviations in LVEDV (−4.48 ± 13.53 ml, *p* < 0.001) and LVESV (−3.28 ± 10.10 ml, *p* < 0.001), the derived parameters LVSV (−1.21 ± 10.63 ml, *p* = 0.035) and LVEF (1.14 ± 4.86 ml, *p* < 0.001) show only minor biases. However, RVEDV (−5.14 ± 13.86 ml, *p* < 0.001) and RVESV (1.60 ± 9.92 ml, *p* = 0.003) deviate in opposite directions, which increases the bias for RVSV (−6.75 ± 12.47 ml, *p* < 0.001) and RVEF (−2.48 ± 4.95 ml, *p* < 0.001). The assessment of LVM revealed an overall small negative bias (−1.82 ± 15.47 g, *p* = 0.028). Mean differences for native T1 (−1.64 ± 11.13 ms, *p* = 0.014) and T2 parametric mapping (0.14 ± 1.06 ms, *p* = 0.101) were close to zero.

#### Clinical acceptability via tolerance ranges

**Fig. 5:**
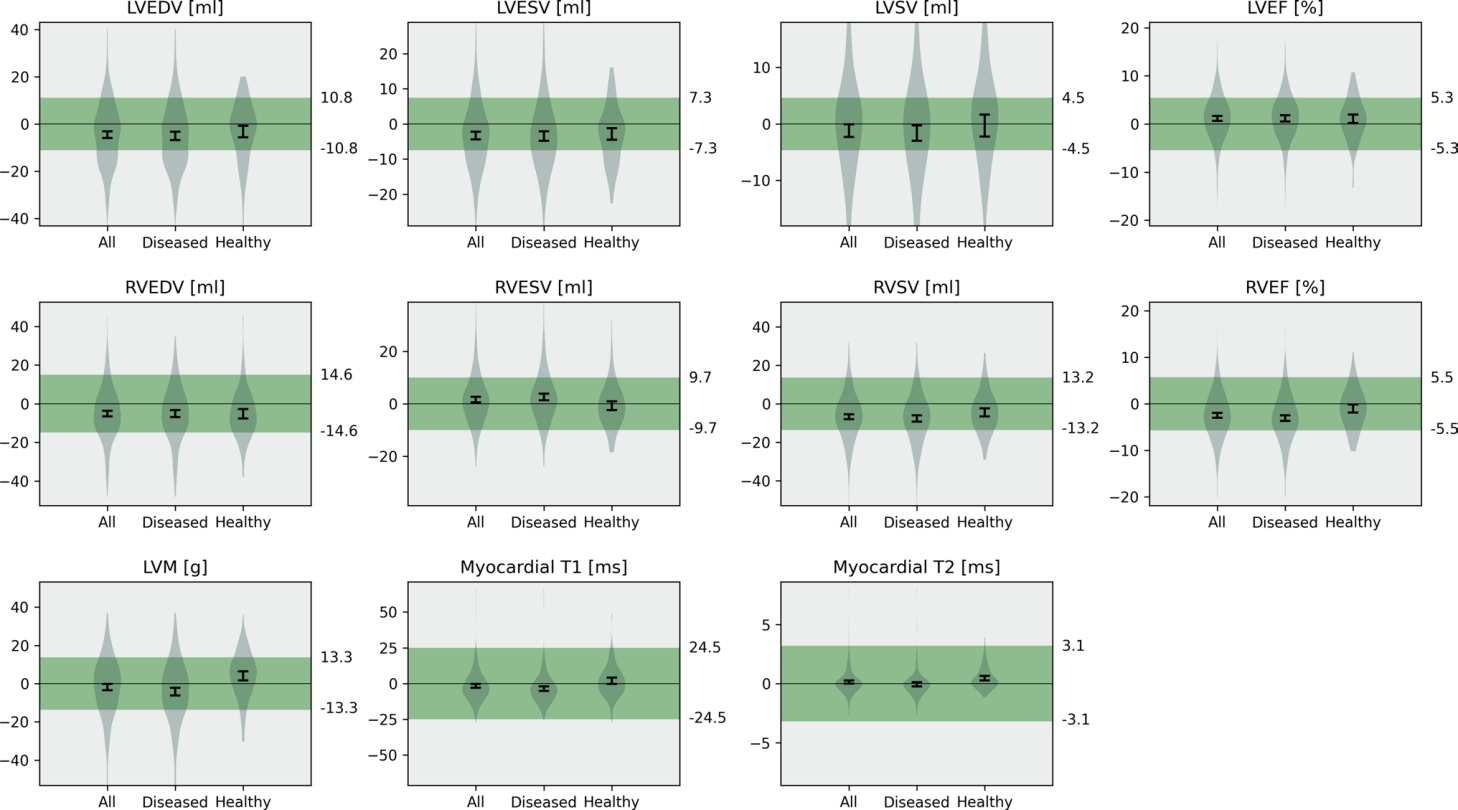
Acceptance testing for clinical parameters. The 95% confidence intervals (black error bar) of mean errors in quantitative clinical parameters are plotted against their respective tolerance intervals (green) based on intraobserver variability. Error bars are provided for the comparison of all cases, as well as diseased and healthy groups. A bias is considered acceptable if its confidence interval lies completely within the tolerance range. LVEDV: left ventricular end-diastolic volume; LVESV: left ventricular end-systolic volume; LVSV: left ventricular stroke volume; LVEF: left ventricular ejection fraction; RVEDV: right ventricular end-diastolic volume; RVESV: right ventricular end-systolic volume; RVSV: right ventricular stroke volume; RVEF: right ventricular ejection fraction; LVM: left ventricular mass

Acceptance testing for differences between Nick and GS showed that the 95% confidence intervals for the mean differences of all parameters were inside their respective tolerance ranges in the overall cohort, as well as in the diseased and healthy subgroups (Fig. 5).

#### Healthy and diseased group analysis

**Fig. 6:**
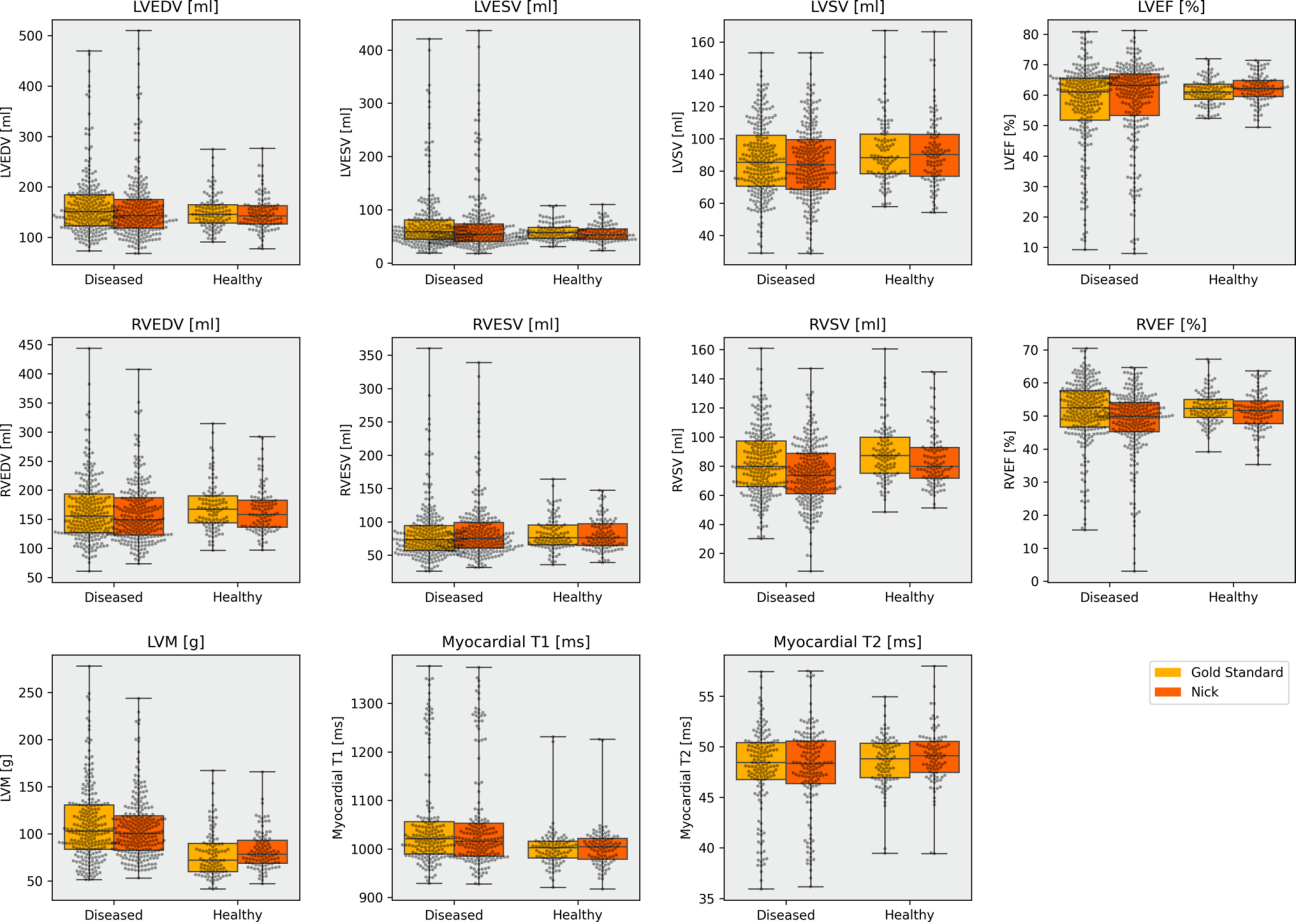
Paired boxplots for quantitative parameters in the healthy and diseased groups. LVEDV: left ventricular end-diastolic volume; LVESV: left ventricular end-systolic volume; LVSV: left ventricular stroke volume; LVEF: left ventricular ejection fraction; RVEDV: right ventricular end-diastolic volume; RVESV: right ventricular end-systolic volume; RVSV: right ventricular stroke volume; RVEF: right ventricular ejection fraction; LVM: left ventricular mass

Table 3 demonstrates mean deviations of Nick and the GS in the healthy and diseased groups. Overall, biases were comparable in both groups with few exceptions: Notable differences were observed for LVM (healthy: 4.00 ± 12.24 g; diseased: −4.25 ± 16.03 g), RVESV (healthy: −0.77 ± 8.55 ml; diseased: 2.59 ± 10.30 ml) and native T1 (healthy: 1.83 ± 10.82 ms; diseased: −3.46 ± 10.87 ms). All other parameters show similar biases for both groups. Paired boxplots for the quantitative parameters derived from Nick and GS segmentations are shown side by side for healthy probands and diseased patients in Fig. 6. Nick and GS share almost identical medians and interquartile ranges, suggesting a comparable distribution of clinical parameter assessments. As an example, it is apparent that the distribution for LVM parameters in the healthy group differs considerably from the diseased group in both the manual and the AI evaluation.

**Table 3:** Comparison of Nick and GS for quantitative clinical parameters across healthy and diseased groups

	Healthy mean ± SD (Nick - GS)	Diseased mean ± SD (Nick - GS)
LVEDV [ml]	−3.15 ± 12.57	−5.04 ± 13.89
LVESV [ml]	−2.86 ± 8.28	−3.45 ± 10.78
LVSV [ml]	−0.29 ± 10.01	−1.59 ± 10.87
LVEF [%]	1.07 ± 4.39	1.16 ± 5.05
LVM [g]	4.00 ± 12.24	−4.25 ± 16.03
RVEDV [ml]	−5.20 ± 12.53	−5.12 ± 14.40
RVESV [ml]	−0.77 ± 8.55	2.59 ± 10.30
RVSV [ml]	−4.44 ± 10.72	−7.71 ± 13.04
RVEF [%]	−1.04 ± 4.35	−3.09 ± 5.07
T1 global [ms]	1.83 ± 10.82	−3.46 ± 10.87
T2 global [ms]	0.45 ± 0.92	−0.06 ± 1.09

GS: gold standard; SD: standard deviation; LVEDV: left ventricular end-diastolic volume; LVESV: left ventricular end-systolic volume; LVSV: left ventricular stroke volume; LVEF: left ventricular ejection fraction; LVM: left ventricular mass; RVEDV: right ventricular end-diastolic volume; RVESV: right ventricular end-systolic volume; RVSV: right ventricular stroke volume; RVEF: right ventricular ejection fraction.

### Subgroup analysis

Subgroup analysis of Nick’s performance in patients with LVH and DCM assessed differences for LVEF, LVSV, RVEF, and RVSV. Left ventricular parameters were underestimated in patients with LVH (LVEF: −1.59 ± 8.49%; LVSV: −9.29 ± 16.13 ml) and slightly overestimated in patients with DCM (LVEF: 1.02 ± 4.06%; LVSV: 3.60 ± 11.47 ml), showing overall good agreement between Nick and the GS. A more pronounced underestimation was observed for RVEF (LVH: −5.46 ± 4.92%; DCM: −3.94 ± 6.29%) and RVSV (LVH: −12.20 ± 13.23 ml; DCM: −11.61 ± 17.21 ml) across both subgroups.

#### Quantification of manual correction workload

**Fig. 7:**
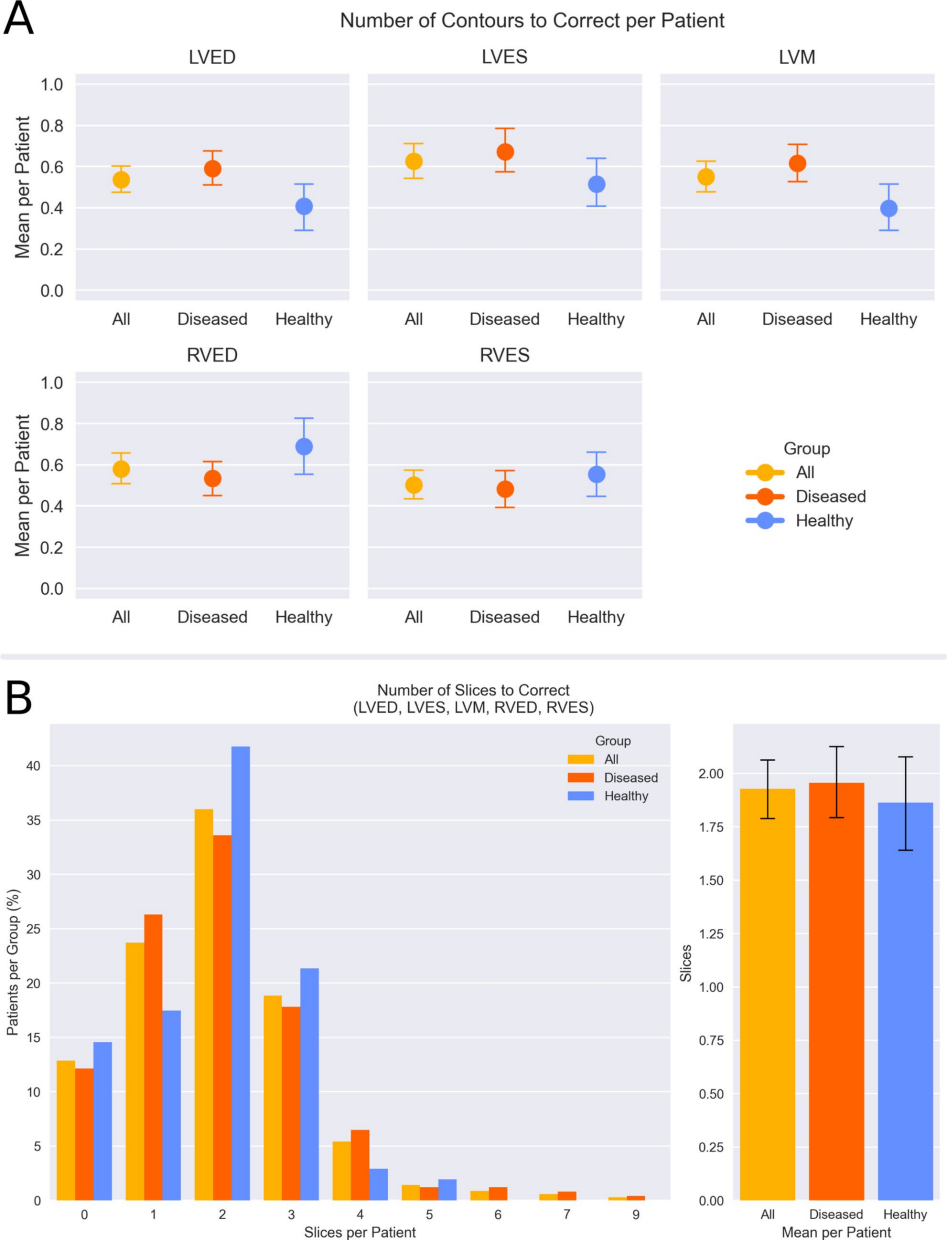
(A) Mean number of contours requiring correction per patient, stratified by contour type and group. (B) Distribution (left panel) and mean (right panel) of the number of slices requiring correction per patient. LVED: left ventricular end-diastolic endocardial segmentation; LVES: left ventricular end-systolic endocardial segmentation; LVM: left ventricular myocardial segmentation; RVED: right ventricular end-diastolic endocardial segmentation; RVES: right ventricular end-systolic endocardial segmentation

In order to evaluate the number of contours in need of manual correction when using Nick, per-slice tolerance ranges for individual contours were defined based on inter-observer variability (see Methods, “quantification of manual correction workload”), resulting in the following threshold values: absolute area difference ≤ 5.6 cm^2^ or DSC ≥ 90% for the LV endocardial segmentation at ED; absolute area difference ≤ ± 4.0 cm^2^ or DSC ≥ 83% for the LV endocardial segmentation at ES; absolute area difference ≤ ± 5.0 cm^2^ or DSC ≥ 71% for the LV myocardial segmentation; absolute area difference ≤ ± 7.4 cm^2^ or DSC ≥ 82% for the RV endocardial segmentation at ED; and absolute area difference ≤ ± 5.5 cm^2^ or DSC ≥ 74% for the RV endocardial segmentation at ES. The mean number of contours requiring correction per case was below 1.0 for all contour types and groups (Fig. 7A). A SAX slice was determined to require correction if at least one of the contours (logical OR) in it exceeded the tolerance cutoffs. On average, fewer than two slices per case required correction in a SAX cine stack (Fig. 7B).

## Discussion

Nick demonstrated robust performance in estimating clinical parameters across different cardiac phenotypes and shows great potential to minimize manual segmentation workload. On average, fewer than two slices per case needed to be corrected in a short-axis stack. In particular, we did not observe nonsensical segmentations and Nick’s selections for ED and ES were overall in agreement with the expert. Acceptance testing for mean differences showed unobjectionable deviations for all clinical parameters.

### Performance across subgroups

Nick’s performance was consistent across healthy volunteers and diseased patients, including cardiac phenotypes such as LVH and DCM. This was particularly clear in boxplot comparisons, where Nick reproduced the parameter distributions of the GS in quantile range, as well as overall group variance. The mean deviations of Nick and GS showed very consistent results in both groups. However, results also highlight the importance of manual review to ensure clinical accuracy. Overall, the bias between Nick and GS in all parameters was moderate and comparable in both groups, indicating that Nick performed similarly well in healthy and diseased individuals – supporting its robustness and generalizability. Acceptability was confirmed using tolerance ranges which reflect the expected clinical variability and were passed by Nick. Nonetheless, there was a bias observed in LVM and RVESV measurements. Global native T1 also exhibited juxtaposed biases in healthy participants (positive) and diseased patients (negative). The observed differences between healthy and diseased groups may be related to regions with higher T1 values in diseased patients, where even small contour changes can noticeably affect the values [24].

The Bland-Altman analysis showed minimal bias and satisfactory limits of agreement for most parameters. Observed biases are likely due to variability in the AI’s segmentation of the basal slices. This particularly concerns the RV parameters and agrees with previous studies where the automated segmentation of the RV is challenging not only because of the shape but also variability of delineation in basal short-axis slices [16, 25]. Generally, most discrepancies were observed in basal slices of both ventricles. These regions are anatomically complex, often showing more variability in delineation due to the oblique position of the outflow tracts, also affected by partial volume artifacts as well as reduced contrast. For LVM, Bland-Altman and qualitative analyses suggest that Nick overestimated smaller (thinner) myocardia and underestimated larger (thicker) myocardia. Nevertheless, the mean number of contours requiring correction per case was below 1.0 for all contour types and groups. In addition, our findings show that Nick consistently identified ED and ES phases that closely aligned with expert selections.

In general, this study has shown that Nick is able to provide accurate, reproducible, and generalizable assessments of cardiac function and parametric mapping in a heterogeneous, multi-centre cohort. This agrees with the existing literature [17, 25, 26], in which convolutional neural network-based AI solutions have been shown to deliver close-to-expert clinical parameters, and generalize across diseases when trained to accommodate diverse datasets.

### Dataset compilation

Our study focused on AI generalizability by including diagnosed patients from multiple sites and evaluating biventricular segmentation tasks for cardiac function and parametric mapping at once. In Bai et al. [27], a strong AI performance was demonstrated using approximately 5,000 healthy participants from the UK Biobank, while our study demonstrated AI performance in healthy volunteers, as well as diagnosed subgroups. Further, we composed a multi-centre dataset to reflect Nick’s capability across sites in routine clinical practice. In contrast, while previous studies have primarily focused on either function or mapping analysis [17, 28, 29], our study provides a comprehensive evaluation of AI performance in both.

### Manual correction workload

We defined segmentation cutoffs based on inter-reader variability to quantify expert disagreement at the contour level, and tolerance ranges for clinical parameters to reflect acceptable variability in derived clinical parameter. Although related, these thresholds address complementary aspects of performance: segmentation deviations do not translate directly into parameter deviations, as errors may either cancel out or accumulate when deriving volumes. In practice, large contour discrepancies were rare (affecting < 1 slice per case and contour type on average), indicating a low manual correction workload when using Nick. In addition, all comparisons of derived clinical parameters remained within their predefined tolerance ranges. Segmentation cutoffs were not applied to parametric mapping, as these measurements are highly sensitive to small, localized pixel boundary errors that are insufficiently captured by segmentation metrics. Instead, the clinical acceptability of mapping segmentations was assessed directly by verifying that all derived relaxation time comparisons remained within their predefined tolerance ranges.

Overall, the mean number of SAX slices needing corrections was below two in either group (all, healthy, diseased). Therefore, while AI-based segmentations should still be manually reviewed, Nick may serve as a time-saving tool in clinical routine.

### Limitations

While patients with diverse cardiac morphologies were included, the study did not include cases with congenital heart disease. Although the dataset incorporates multiple sites and field strengths, input images were acquired with scanners from only one vendor. The evaluation focussed on expert-AI comparison and did not include a scan-rescan assessment to quantify parameter variability across repeated acquisitions.

## Conclusion

Nick demonstrated strong correlations to the expert in both healthy and diseased groups, with comparisons for mean deviations in all clinical parameters lying within their respective tolerance ranges. Overall, the AI-generated contours required only few manual corrections when compared to an expert. Nick may be used as a support tool in clinical routine with minimal human intervention.

## Supplementary Information

Below is the link to the electronic supplementary material.

Supplementary Material 1

## Data Availability

The dataset analysed in this study is not publicly available due to German legislation but may be obtained upon reasonable request to the corresponding author.

## Abbreviations

AI: Artificial intelligence
CMR: Cardiovascular magnetic resonance
DCM: Dilated cardiomyopathy
DSC: Dice similarity coefficient
ED: End-diastole
EDV: End-diastolic volume
EF: Ejection fraction
ES: End-systole
ESV: End-systolic volume
GS: Gold standard
LAX: Long axis
LV: Left ventricle
LVH: Left ventricular hypertrophy
LVM: Left ventricular mass
RV: Right ventricle
SAX: Short axis
SCMR: Society for cardiovascular magnetic resonance
SV: Stroke volume
