# Supplementary material for: Assessing the robustness of an artificial intelligence segmentation model for quantitative cardiovascular magnetic resonance imaging across cardiac phenotypes
Source: Int J Cardiovasc Imaging. 2025 Dec 26;42(2):343–57. doi: 10.1007/s10554-025-03596-3 (PMC12909472; doi:10.1007/s10554-025-03596-3)
Supplement: Supplementary file 1 — Supplementary Material 1 [file 10554_2025_3596_MOESM1_ESM.pdf]

Supplementary Information

Network training

For short-axis segmentation, an initial baseline algorithm was pretrained on 3,000 cardiovascular magnetic resonance (CMR) studies (10% reserved for validation) and tested on 1,719 unseen cases (22,640 individual images) from the UK Biobank (application 2964) and on 700 studies from the Second Annual Data Science Bowl Kaggle challenge, with 200 additional studies for validation and 440 for testing. The baseline model was then finetuned on 1,124 consecutively acquired clinical CMR studies, with 940 used for training, 90 for validation, and 94 for testing.

For segmentation of T1 and T2 mapping images, we used a pretrained Dense U-Net [1] which was further trained on 3,095 T1 maps and 1,210 T2 maps, together with the corresponding T1- and T2-weighted images from motion-corrected source images. Data were collected from 539 subjects (458 training, 33 validation, 48 testing). In the test subset, only the T1 and T2 maps were included. As for cine data, post-processing retains only the largest connected component for the left ventricular myocardium in each 2D map.

Supplementary Table 1: Clinical diagnoses in the diseased subcohort

Diagnosis	Number
Atrial fibrillation	3
Amyloidosis*	2
Arrhythmogenic right ventricular cardiomyopathy	1
Coronary artery disease	20
Dilated cardiomyopathy (DCM)	36
Hypertrophic cardiomyopathy (HCM)*	30
Hypertension	12
Ischemic heart disease	8
Inflammatory heart disease	49
Muscular dystrophy	3
Psoriasis	59
Valvular heart disease	32

*Left ventricular hypertrophy (LVH) includes amyloidosis and hypertrophic cardiomyopathy (HCM)

References

1. Popescu AB, Seitz A, Mahrholdt H, et al (2025) Deep learning-based segmentation of T1 and T2 cardiac MRI maps for automated disease detection. https://doi.org/10.48550/ARXIV.2507.00903
